# Life Cycle Assessment of the Sustainability of Alkali-Activated Binders

**DOI:** 10.3390/biomimetics8010058

**Published:** 2023-02-01

**Authors:** Mohammad Alhassan, Ayah Alkhawaldeh, Nour Betoush, Mohammad Alkhawaldeh, Ghasan Fahim Huseien, Layla Amaireh, Ahmad Elrefae

**Affiliations:** 1 Civil Engineering Program, College of Engineering, Al Ain University (AAU), Al Ain 64141, United Arab Emirates; 2 Department of Civil Engineering, College of Engineering, Jordan University of Science and Technology (JUST), Irbid 21110, Jordan; 3 Department of Civil Engineering, College of Engineering, Al-Ahliyya Amman University, Amman 19328, Jordan; 4 Department of the Built Environment, College of Design and Engineering, National University of Singapore, Singapore 117566, Singapore; 5 Department of Civil, Materials, and Environmental Engineering, College of Engineering, University of Illinois at Chicago (UIC), Chicago, IL 60607, USA

**Keywords:** alkali-activated binders, carbon dioxide, embodied energy, geopolymer concrete, greenhouse, LCA, sustainability, TOPSIS

## Abstract

Limiting the consumption of nonrenewable resources and minimizing waste production and associated gas emissions are the main priority of the construction sector to achieve a sustainable future. This study investigates the sustainability performance of newly developed binders known as alkali-activated binders (AABs). These AABs work satisfactorily in creating and enhancing the concept of greenhouse construction in accordance with sustainability standards. These novel binders are founded on the notion of utilizing ashes from mining and quarrying wastes as raw materials for hazardous and radioactive waste treatment. The life cycle assessment, which depicts material life from the extraction of raw materials through the destruction stage of the structure, is one of the most essential sustainability factors. A recent use for AAB has been created, such as the use of hybrid cement, which is made by combining AAB with ordinary Portland cement (OPC). These binders are a successful answer to a green building alternative if the techniques used to make them do not have an unacceptable negative impact on the environment, human health, or resource depletion. The Technique for Order Preference by Similarity to Ideal Solution (TOPSIS) software was employed for choosing the optimal materials’ alternative depending on the available criteria. The results revealed that AAB concrete provided a more ecologically friendly alternative than OPC concrete, higher strength for comparable water/binder ratio, and better performance in terms of embodied energy, resistance to freeze–thaw cycles, high temperature resistance, and mass loss due to acid attack and abrasion.

## 1. Introduction

The first documented attempt for developing geopolymer concrete, which is also referred to as alkali-activated binder (AAB) concrete, was reported in 1939–1940 according to the historical review papers published by Li et al. [1] and Pacheco-Torgal et al. [2]. In terms of lime and ordinary Portland cement (OPC), this new form of binder is designated as a third cement generation [1,2]. New types of binders are always encouraged for the development and improvement of durability of concrete, strength, and most importantly environmental preservation. These novel binders are based on the notion of employing ashes from mining and quarrying wastes as raw materials in the treatment of hazardous and radioactive wastes [3,4]. The study by Purdon in 1940 [5] contributed significantly to the creation of AAB concretes, which gained popularity during the 1940s. Feret’s work [6] is considered relevant to the area of geopolymer concrete; however, it is mainly about the use of slag in OPC concrete as supplementary cementitious material. Purdon [5] employed blast furnace slag activated with sodium hydroxide in two phases, first releasing silica, aluminum, and calcium hydroxide. The development of silica and aluminum hydrates followed, as did the renovation of the alkali solution. Davidovits (1994) [7] created an alkali binder by activating metakaolin binders called geopolymers or polysialates. This high alkali cement was created using an inorganic polycondensation reaction known as geopolymerization. The polysialate network is made up of tetrahedral anions (-Si-O-Al-O). Geopolymers have strong mechanical performance as well as an instantly recognizable property indicated by high early strength, minimal shrinkage, superior acid, and abrasion resistance, preferred freeze–thaw cycle resistance, and exceptional resistance to fire and high temperatures [7,8].

In a research paper, the fracture behavior of geopolymer composites made of fly ash or metakaolin, fine aggregate, and river sand and reinforced with glass, carbon, and aramid fiber was examined at three different temperatures, around 3 °C, 20 °C, and 50 °C. As a potential working temperature for composite materials created using additive manufacturing technology, the temperatures were considered. Bending strength testing was the primary study methodology employed. The results revealed that adding fibers enhanced the bending strength of all composites substantially. The metakaolin-based composites and the sand reinforced with 2% of weight aramid fiber produced the greatest results at room temperature. The results at 50 °C revealed a considerable drop in bending strength for virtually all compositions, which is surprising given that geopolymers are advertised as materials designed to perform at high temperatures. For nearly all compositions, the test at low temperature (about 3 C) revealed an increase in bending strength [9].

Several investigations have proven that acceptable geopolymer behavior may also be produced by employing industrial wastes as secondary source material, such as fly ash or slag. The environmental effect of geopolymer manufacturing is dependent on the type of raw material generated under different circumstances to attain different features [10]. The AABs are considered sustainable materials as a result of their great durability and much lower carbon dioxide emissions than OPC. According to studies, the CO_2_ intensity of these binders is roughly 2.4 times lower than that of OPC [11,12]. The sustainability elements of geopolymers are connected to their high environmental advantages as a result of their wonderful qualities in terms of thermal stability and greenhouse gas emission [13,14,15]. In order to produce more environmentally friendly and sustainable construction materials, the use of high-volume fly ash (FA) as a cement alternative in the manufacturing of concrete has become valuable [16]. The usage of geopolymer concrete is being considered as a possible substitute for traditional concrete and as a way to transform various waste streams into beneficial by-products [17].

One of the most essential criteria for sustainability performance is the life cycle assessment (LCA) of geopolymer concrete, which analyzes the ecological effect and environmental potential of materials based on the stages of production and manufacture, usage, and demolition. The LCA is divided into four stages: aim and scope definition, inventory analysis, impact assessment, and interpretation [18,19]. A life cycle assessment technique is used to calculate mechanical strength, durability, energy, cost, and emissions criteria. By providing a complete depiction of the needed embodied energy and CO_2_ emissions in feedstocks, this technique enables a thorough comparison of material production, transit, usage, and demolition consequences. The life cycle phases for material feedstock production, such as collection, transportation, mining, and calcination of these feedstocks, are investigated first, followed by manufacturing stages, including the mixing process of these raw materials [20,21].

Another aspect of sustainability is the availability of resources, which plays a significant role and has a major influence on the cost, including the following stages: excavation process, extraction, transportation, forming, and construction time. As a result, the high availability of resources activates the building process and decreases the overall project time in order to reduce purchasing effort while also reducing human potential in the search for alternatives [22,23]. In material selection, the availability of construction materials is viewed as an embodied energy criterion. Furthermore, even if the research location is remote, it is preferable to use locally available materials in the project rather than materials available in remote areas that require time and cost in transportation, in order to achieve environmental protection and reduce gas emissions during transportation and excavation. As a consequence, using accessible building materials reduces excavation potential, cost, gas emissions, working time, human potential, and embodied energy, so that it is enhancing sustainability [24,25].

Based on the current and anticipated momentum toward the use of AAB concrete in the field of sustainability, this study is performed to provide useful life cycle cost comparison between typical AAB and OPC concrete. The aim of this study is to develop effective analysis using the TOPSIS software that is beneficial to the field of green building sustainability. The methodology and interesting results are presented and discussed systematically in the following sections of this paper.

## 2. Performance—Sustainability Characteristics

**Figure 1:**
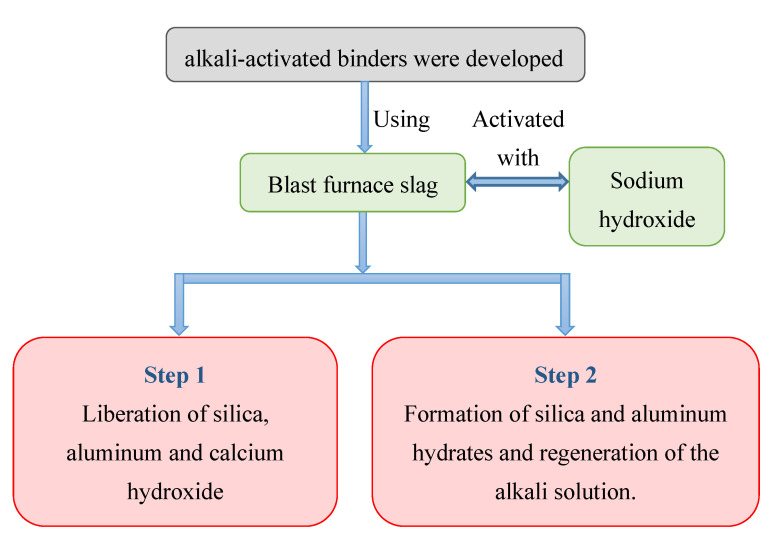
Alkali-activated binders’ first development by Purdon in 1940 [5].

Purdon’s work [5] as well as the invention of AABs made major contributions in the 1940s to the field of sustainability. Purdon made use of sodium hydroxide-activated blast furnace slag. The process was developed in two stages, according to the author. Silica, calcium hydroxide, and aluminum were liberated in the first. The alkali solution would then be regenerated, and silica and aluminum hydrates would form (Figure 1). The AAB concrete outperforms OPC concrete in terms of both mechanical performance and acid and abrasion resistance. Several approaches are employed in estimating sustainability performance analysis for different types of concretes. The LCA method, preference selection index (PSI) method, and TOPSIS method were all utilized in this study in order to compare the performance of AAB concrete with the performance of the OPC concrete in terms of several aspects including: environmental effects, mass loss due to acid attack, mass loss due to abrasion, embodied energy, number of freeze–thaw cycles, and sustained temperature. The LCA method is widely accepted as a systematic approach for assessing the environmental performance of concrete production process across its entire life cycle [21]. The LCA allows for balancing the materials and energy consumptions while measuring the broad environmental impact. The PSI technique is becoming very beneficial when there is ambiguity regarding comparative characteristics. Using the overall preference value, the PSI for each option is computed, and the optimum alternative with the maximum PSI value is selected [26]. The core principle behind the use of the TOPSIS technique is that the best choice must be the one that is the closest to the ideal solution and the farthest from the negative-ideal solution. The performed analysis using the here aforementioned approaches is described in the following sections.

### 2.1. Mechanical Strength

AAB concrete frequently has greater tensile and flexural strengths than its compressive strength when compared to OPC concrete. It is hypothesized that this would strengthen the fracture resistance of geopolymer concrete since it seems that the geopolymer gel is strongly bound to the aggregate particles [7,27]. The relationship between the tensile and compressive strengths of the AAB concrete at 28 days of curing was established using a nonlinear mathematical model [28]. It was found that the tensile-to-compressive strength ratio decreases as the compressive strength increases. The splitting tensile strength (*f_t_*) and compressive strength (*f*′*_c_*) do not directly relate according to Power’s law, given as: *f_t_* = *k(f*′*_c_)*^0.5^ [29]. It was suggested to use a straightforward power function, *f_t_* = 0.249 *f*′*_c_*^0.772^, to calculate the splitting tensile strength from the compressive strength of AAB concretes regardless of the grade or molarity. This proposed formula shows good agreement with experimental results [28].

### 2.2. Durability

One of the major benefits of AAB concrete over OPC concrete is their high resistance to acid attack. The OPC concrete has a typical mass loss after exposure to acid attack of approximately 70–95% compared to approximately 8–12% for the AAB concrete under the similar exposure condition. This indicates that the AAB concrete experiences a lower mass loss of approximately 88% [30,31]. Regarding the resistance to high temperatures, the OPC concrete demonstrates weak performance and disintegrates when the temperature exceeds 300 °C, whereas the AAB concrete typically exhibits outstanding stability even up to 1000 °C [32]. In addition, the AAB concrete has a robust fire resistance, which is a necessary characteristic for use in projects with potential exposure to fire such as in tunnels or large structures [33]. For a companion compressive strength, the resistance to freeze–thaw cycles of the AAB concrete is about twice the OPC concrete [34]. In terms of the resistance to abrasion measured as mass loss, the OPC concrete experiences approximately 45%–60% compared with approximately 15–25% for the AAB concrete [35].

### 2.3. Embodied Energy and CO_2_ Emissions

Manufacturing of Portland cement is thought to be responsible for approximately 8% of the world’s CO_2_ emissions, which is the greatest among all building material. On average, 0.81 kg of CO_2_ is produced for every 1 kg of cement during the Portland cement clinker manufacturing including the calcination process [36]. From a sustainability perspective, geopolymer concrete should only be considered as a green construction alternative if the manufacturing processes used to make it do not adversely damage the environment, deplete resources, or have a detrimental impact on human health. Geopolymer concrete used to have a CO_2_ emission that was almost 10% lower than comparable concrete with a 100% OPC binder [12]. A recent study evaluated the viability of employing industrial wastes for geopolymer 3D printing in factories and building sites to provide a deep understanding of the construction cost, time, and energy consumption [37]. The study revealed that the use of sustainable materials by dependable and inexpensive manufacturing procedures in the building sector cuts the construction time and lowers the energy demand by about 50% compared with traditional manufacturing techniques. The cost of producing a 3D-printed house is around 32% lower than the cost of producing a building using OPC and traditional manufacturing methods. As a replacement for OPC concrete in the building sector, an AAB concrete makes a positive contribution toward reducing the CO_2_ emissions and global warming [37].

### 2.4. Cost

In the designing of greenhouses, AAB concrete is shown to be much better than OPC and might be cost-competitive on a production-only basis. According to prime materials, the cost of ordinary OPC structural concrete is roughly 125 dollars per ton, but the cost of AAB structural concrete for the same specifications is between 118–175 dollars per ton [17]. The main reason for the fluctuation in the price of the AAB concrete can be attributed to the fact that most scientific studies focus on the utilization of fly ash and slag materials that are becoming limited or becoming more expensive as a result of environmental legislation and the minimization of coal-fired power generation. Therefore, finding new sources of waste that are not the by-products of coal combustion is becoming necessary [38]. Another issue is attributed to variations in the raw materials cost, especially the sodium hydroxide (one of the primary ingredients of the AAB concrete) that experienced significant fluctuation in its price over the past few years [39].

## 3. Life Cycle Cost Assessment

**Figure 2:**
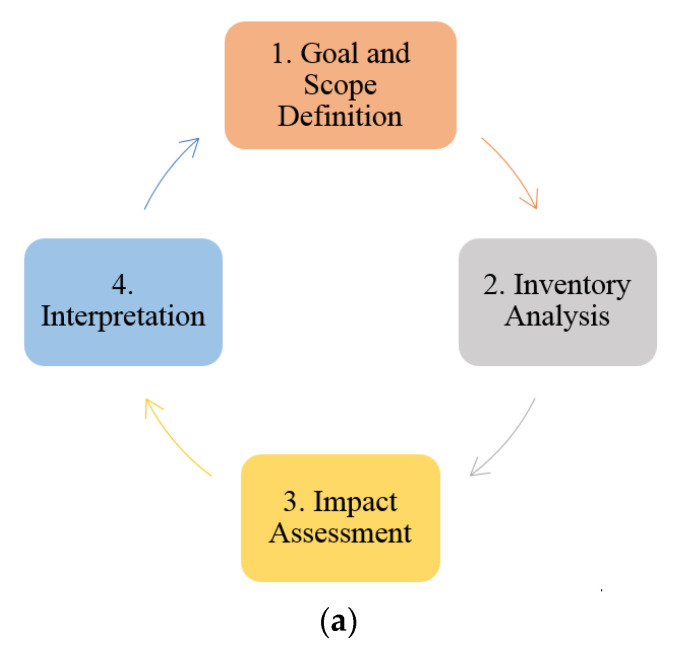

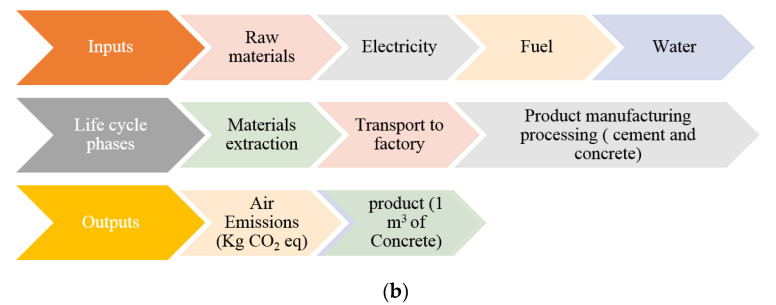
Phases of life cycle assessment. (**a**) Life cycle assessment framework. (**b**) Inputs and outputs in life cycle assessment.

**Figure 3:**
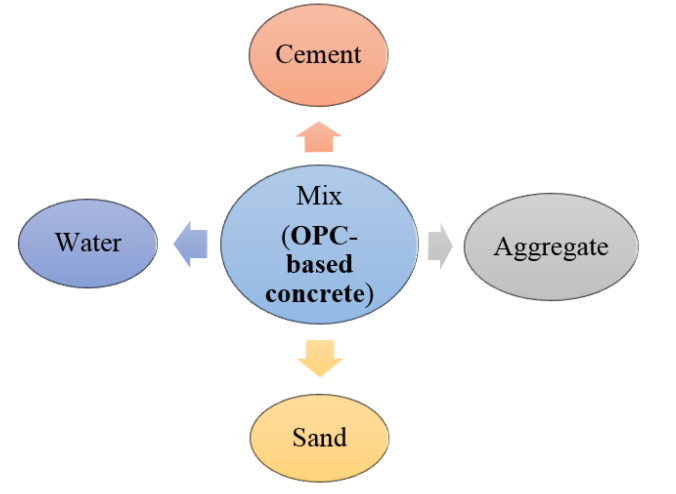
Schematic representation of the ingredients of OPC concrete.

**Figure 4:**
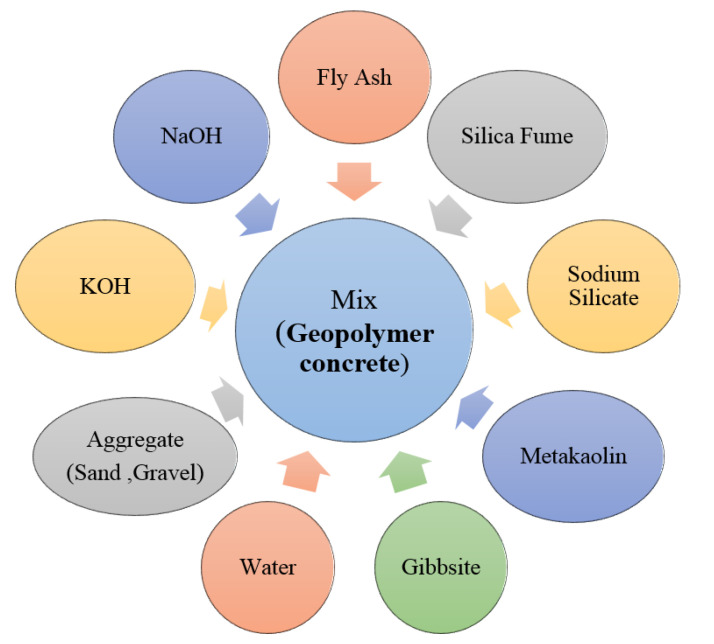
Schematic representation of the ingredients of geopolymer concrete.

A life cycle assessment, or LCA, is a method for calculating the environmental effects associated with the extraction, mining, quarrying, manufacture, usage, and demolition stages of structural buildings [40]. This section represents a comparison between the life cycle assessment of the AAB concrete and the OPC concrete. A general representation of life cycle phases applied in the production of concrete from cradle to gate are represented in Figure 2. The ingredients used in the production of the OPC and AAB concretes are shown in Figure 3 and Figure 4, respectively.

**Figure 5:**
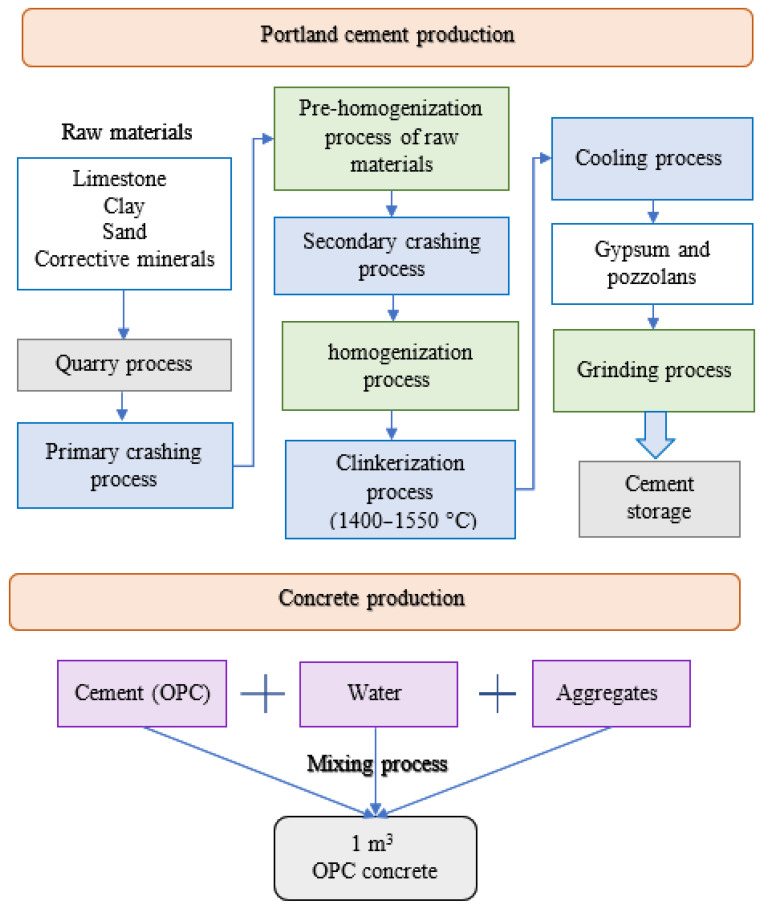
Life cycle stages from cradle to gate for OPC concrete production.

**Figure 6:**
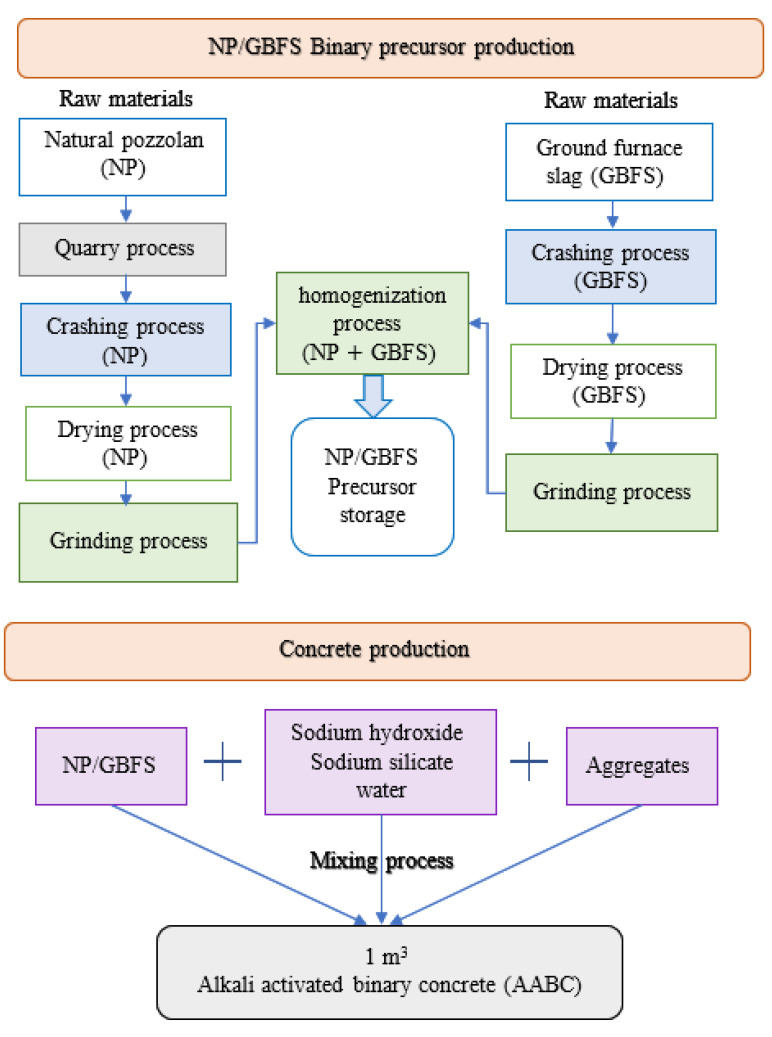
Life cycle stages from cradle to gate for AABC concrete production.

**Figure 7:**
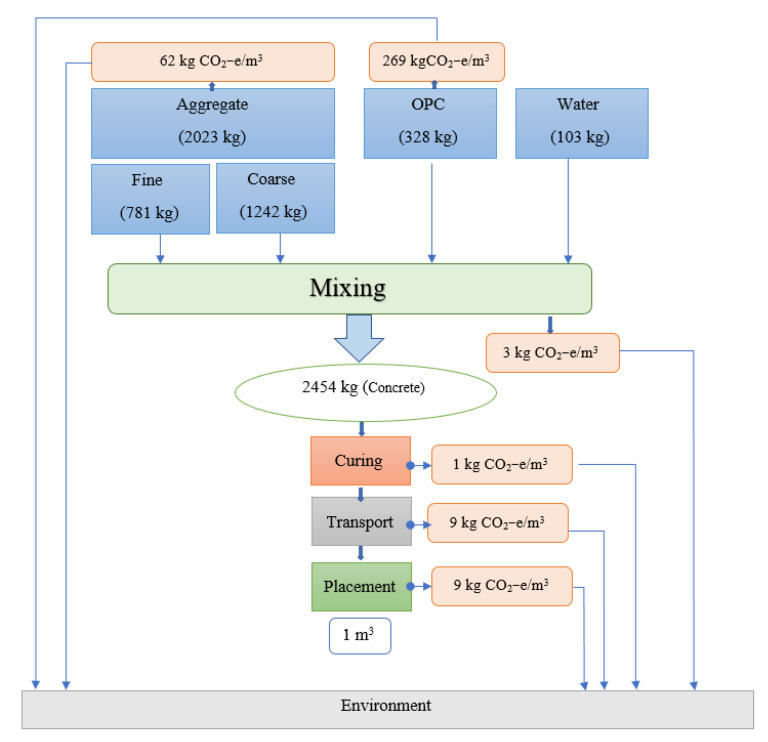
Life cycle stages for OPC concrete.

**Figure 8:**
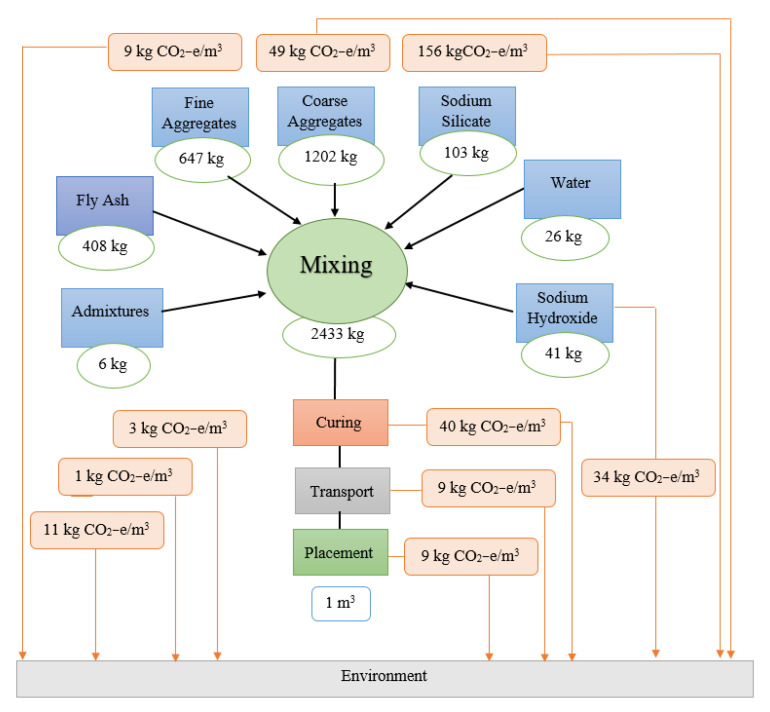
Life cycle stages for alkali-activated binder (geopolymer) concrete.

The production stages from cradle to gate of the OPC concrete and the AAB concrete [19] are represented in Figure 5 and Figure 6, respectively. The process of producing OPC concrete is illustrated in Figure 5 at each stage from the extraction of the limestone through the clinkerization, grinding, and manufacturing. Moreover, typical manufacturing process of the AAB concrete is shown in Figure 6, which includes the extraction procedures and preparation of raw materials such as natural pozzolan (NP) and ground blast furnace slag GBFS, homogenization, and the addition of aggregates and alkaline activators. A comparison between the OPC concrete and the AAB concrete was created by constructing the life cycle stages of each and then computing the CO_2_ emission. To compute the CO_2_ emission and construct the life cycle stages, a concrete with 40 MPa compressive strength was studied for the two binder types according to the mixing proportions shown in Table 1 [12]. The life cycle stages for producing 1 m^3^ of OPC concrete and AAB concrete are displayed in schematic graphs shown in Figure 7 and Figure 8, respectively. The numbers shown in Figure 7 represent the amount of CO_2_ emissions from the OPC concrete manufacturing, transportation from the cement factory to the ready-mix plant, aggregate extraction and transportation, mixing of the concrete ingredients, curing, and finally placement (i.e., concrete casting). Figure 8 presents the amount of CO2 emissions originated from producing the AAB material, preparation of the concrete mix, curing, transportation, and placement of the AAB concrete. The life cycle stages for the OPC concrete and AAB concrete show that the CO_2_ emission for the AAB concrete is lower than that of the OPC concrete by approximately 9% for each 1 m^3^, as presented in Table 2 [12], which indicates that the use of AAB concrete is more favorable than the OPC concrete in greenhouse construction.

## 4. Application of Preference Selection Index (PSI) Method

**Figure 9:**
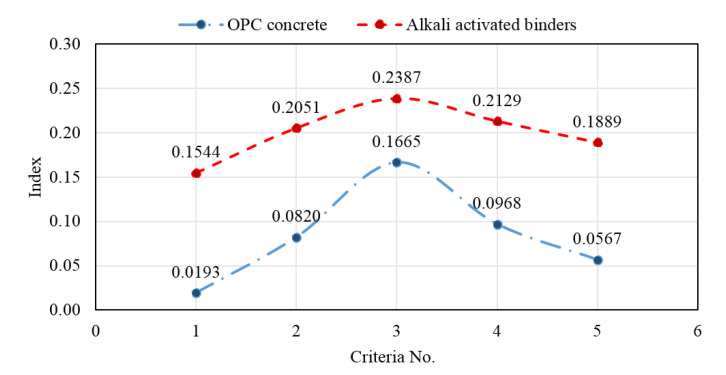
Selection index based on the criteria.

The PSI technique follows a systematic scientific approach to identify the optimum material for a given job. It is considered very useful when there is concern about the relative significance of aspects. The PSI for each option is computed using the overall preference value and the option with the maximum PSI value is chosen as the best alternative. The following calculations are an application of the PSI method [26] to compare OPC concrete and AAB concrete; they are used as an alternative. The classification criteria are as follows: acid attack mass loss, abrasion mass loss, embodied energy, freeze–thaw cycle resistance, and high temperature resistance. The most suitable values for the used criteria are: minimum mass loss due to acid attack and abrasion resistance; minimum embodied energy; maximum endurance to freeze–thaw cycles; and maximum resistance to high temperatures. These criteria are collected from the literature [41] and summarized in Table 3. The PSI formulae are shown in the following representative steps. The PSI data and results are shown in Table 3, Table 4, Table 5, Table 6 and Table 7 and in Figure 9.

Step 1: Compute the normalized (*R_ij_*)

(1) $$R_{ij}=\frac{x_{j}{}^{min}}{x_{ij}}$$

(2) $$R_{ij}=\frac{x_{ij}}{\;x_{j}{}^{max}}$$

where *x_ij_* is the criteria for comparison, if the expectancy is the min-the-better, then Equation (1) should be used, whereas if the expectancy is the max-the-better, then Equation (2) should be used. The computation of the normalized (*R_ij_*) is illustrated in Table 4.

Step 2: Compute preference variation value (*PV_j_*)

(3) $$PV_{j}=\overset{N}{\underset{i=1}{\sum }}{\left[R_{ij}-{\bar{R}}_{j}\right]}^{2}$$

(4) $${\bar{R}}_{j}=\frac{1}{N}\overset{N}{\underset{i=1}{\sum }}R_{ij}$$

Table 5 presents the calculated *PV_j_* values of each criterion used for the comparison between the OPC and AAB concretes. Then, the summation of *PV_j_* is measured.

Step 3: Determine overall preference value (Ψ*_j_*)

(5) $$\Phi_{j}=1-PV_{j}$$

(6) $$\Psi_{j}=\frac{\Phi_{j}}{\sum_{j=1}^{M}\Phi_{j}}$$

(7) $$\underset{j}{\sum }\Psi_{j}=1$$

The value of Ψ*_i_* for each criterion was measured using Equations (5) and (6) and is presented in Table 6. Equation (7) was used as a check for calculations since the sum of Ψi for all criteria should be equal to one.

Step 4: Compute the preference selection index (*I_i_*) for each alternative

(8) $$I_{i}=\overset{M}{\underset{j=1}{\sum }}\left(R_{ij}\times \Psi_{j}\right)$$

The final step is calculating the index *I_i_* for each alternative using Equation (8). The values of *I_i_* are presented in Table 8.

A comparison of *I_i_* between the OPC and AAB concretes is presented in Figure 9. Using the PSI technique for material selection, the best option was a new AAB concrete based on the fact that the index *I_i_* is higher for AAB concrete for all applied criteria: acid attack, abrasion resistance, embodied energy, resistance to freeze–thaw cycles, and resistance to high temperature.

## 5. TOPSIS Model in Material Ranking

A comparison study between alkali-activated binder concrete and OPC concrete has been conducted using TOPSIS software [42] to select the optimal material based on six criteria: minimum mass loss due to acid attack, minimum mass loss due to abrasion, minimum CO_2_ emissions, minimum embodied energy, maximum resistance to freeze–thaw cycles and maximum resistance to high temperature. The core idea behind this strategy is that the chosen alternative should be mathematically closest to the ideal solution and farthest from the negative-perfect solution. The input values shown in Table 8 for the two options were collected from literature [41]. Table 8 summarizes the criteria values, options, and goals based on TOPSIS software. It can be stated that the AAB concrete outperforms OPC concrete according to sustainability performance, and these results from TOPSIS are compatible with the results obtained from the PSI method. Detailed description of TOPSIS functionality that is part of Triptych software is available from the Statistical Design Institute (SDI) for advanced product design.

## 6. Future Trends in AAB Concretes

AAB concretes have the potential to be more ecologically and economically responsible in areas where cement would need to be transported over longer distances and in the event that cement becomes a scarcer resource in the future. In comparison to OPC concretes, the AAB concretes will have a favorable environmental impact. Examples of these types include the use of bricks, one-part geopolymers, and hybrid cements. As an excellent substitute for the clay combustion technique used in the production of burnt bricks, AAB concrete may be used in the form of blocks. In comparison to producing a Ca alumina silicate slag, producing a slag of albite and sodium hydroxide yields a highly reactive solid product with a high alkali content and Si and Al sources that are rapidly liberated in solution. The fundamental idea behind this sort is to combine OPC with AAB, which will reduce the CO_2_ emissions while improving the strength of concrete.

## 7. Conclusions

Based on the results of this study, the following conclusions are drawn.

- The development of AAB concrete seems to provide a more ecologically friendly alternative to OPC concrete. In greenhouse applications, AAB concretes perform more efficiently than OPC concretes.
- According to the PSI technique of material selection, AAB concrete outperforms OPC concrete in the following criteria: mass loss due to acid attack, abrasion resistance, embodied energy, resistance to freeze–thaw cycles, and high temperature resistance.
- In terms of sustainability considerations, AAB concrete is preferable to OPC concrete, as indicated by the PSI technique and TOPSIS.

## Figures and Tables

**Table 1:** Mixture proportions for 40 MPa concrete [12].

Material	Concrete Mixture Proportions (kg/m^3^)	
	OPC Concrete	AAB Concrete
Coarse aggregates	1242	1202
Fly ash	-	408
Fine sand	781	647
Free water	190	26
Superplasticizer	-	6
Sodium hydroxide	-	41 (16 M)
Sodium silicate (Na_2_O = 14.7%, SiO_2_ = 29.4%, water = 55.9%)	-	103
Portland cement	328	-
Curing	Moist curing	Steam curing at 60 °C for 24 h

**Table 2:** Comparison of CO_2_ emissions between OPC and AAB concretes (per 1 m^3^) [12].

Process (i.e., Extraction, Mining, Quarrying, Production, etc.)	CO_2_ Emissions Values (kg/m^3^)	
	OPC Concrete	AAB Concrete
Coarse aggregate	51	49
Fine aggregate	11	9
Portland cement	269	-
Fly ash	-	11
Sodium hydroxide	-	34
Admixtures	-	1
Sodium silicate	-	156
Batching	3	3
Curing	1	40
Transport	9	9
Placement	9	9
Total	353	321

**Table 3:** Quantitative data of material selection attributes [41].

Material	Criteria				
	% Mass Loss Due to Acid Attack	% Mass Loss Due to Abrasion	Embodied Energy (GJ/m^3^)	Freeze–Thaw Cycles (Relative No.)	Sustained Temperature (°C)
OPC concrete	80	50	2.15	1	300
AAB concrete	10	20	1.5	2.2	1000

**Table 4:** Normalized *R_ij_* data of material selection attributes.

Material	Criteria (*x_ij_*)				
	% Mass Loss Due to Acid Attack	% Mass Loss Due to Abrasion	Embodied Energy (GJ/m^3^)	Freeze–Thaw Cycles (Relative No.)	Sustained Temperature (°C)
OPC concrete	0.1250	0.4000	0.6977	0.4545	0.3000
AAB concrete	1.0000	1.0000	1.0000	1.0000	1.0000
Average (*R_ij_*)	0.5625	0.7000	0.8488	0.7273	0.6500

**Table 5:** Preference variation value (*PV_j_*).

Material	Criteria				
	% Mass Loss Due to Acid Attack	% Mass Loss Due to Abrasion	Embodied Energy (GJ/m^3^)	Freeze–Thaw Cycles (Relative No.)	Sustained Temperature (°C)
OPC concrete	0.1914	0.0900	0.0229	0.0744	0.1225
AAB concrete	0.1914	0.0900	0.0229	0.0744	0.1225
Σ(*PV_j_*)	0.3828	0.1800	0.0457	0.1488	0.2450

**Table 6:** Computed overall preference value.

Overall Preference Value	Φ*_i_*	Ψ*_i_*
Acid attack	0.6171875	0.15438461
Abrasion	0.82	0.20511657
Embodied energy	0.95429962	0.23871057
freeze-thaw cycles	0.85123967	0.21293093
high temperature	0.755	0.18885733
∑	3.99772679	1

**Table 7:** Preference selection index for each alternative (*I_i_*).

Material	Criteria					
	% Mass Loss Due to Acid Attack	% Mass Loss Due to Abrasion	Embodied Energy (GJ/m^3^)	Freeze–Thaw Cycles (Relative No.)	Sustained Temperature (°C)	∑*I_i_*
OPC concrete	0.0193	0.0820	0.1665	0.0968	0.0567	0.4213
AAB concrete	0.1544	0.2051	0.2387	0.2129	0.1889	1.0000

**Table 8:** Selection of best alternative based on TOPSIS software.

Criteria	Unit	Option		Goal	−Ideal	+Ideal
		AAB Concrete	OPC Concrete			
Mass loss due to acid attack	%	10	80	minimize	80	10
Mass loss due to abrasion	%	20	50		50	20
CO_2_ emissions	kg/m^3^	320	354		354	320
Embodied Energy	kg/m^3^	1.5	2.15		2.15	1.5
Freeze–thaw cycles	NO.	2.2	1	maximize	1	2.2
Sustained temperature	°C	1000	300		300	1000

## Data Availability

Not applicable.

## References

[1] Li C., Sun H., Li L. (2010). A review: The comparison between alkali-activated slag (Si+Ca) and metakaolin (Si+Al) cements. Cem. Concr. Res..

[2] Pacheco-Torgal F., Castro-Gomes J., Jalali S. (2008). Alkali-activated binders: A review. Part 2. About materials and binders manufacture. Constr. Build. Mater..

[3] Pacheco-Torgal F., Castro-Gomes J., Jalali S. (2008). Alkali-activated binders: A review. Constr. Build. Mater..

[4] van Deventer J.S.J., Provis J.L., Duxson P., Brice D.G. (2010). Chemical Research and Climate Change as Drivers in the Commercial Adoption of Alkali Activated Materials. Waste Biomass Valorization.

[5] Purdon A.O. (1940). The action of alkalis on blast-furnace slag. J. Soc. Chem. Ind..

[6] Feret R. (1939). Slags for the manufacture of cement. Review. Mater. Constr. Tr. Publ..

[7] Davidovits J. Properties of geopolymer cements. Proceedings of the First International Conference on Alkaline Cements and Concretes Scientific Research Institute on Binders and Materials Kiev.

[8] Pacheco-Torgal F., Abdollahnejad Z., Camões A.F., Jamshidi M., Ding Y. (2012). Durability of alkali-activated binders: A clear advantage over Portland cement or an unproven issue?. Constr. Build. Mater..

[9] Korniejenko K., Figiela B., Ziejewska C., Marczyk J., Bazan P., Hebda M., Choińska M., Lin W.-T. (2022). Fracture Behavior of Long Fiber Reinforced Geopolymer Composites at Different Operating Temperatures. Materials.

[10] Weil M., Dombrowski K., Buchwald A. (2009). Life-cycle analysis of geopolymers. Geopolymers.

[11] Yang K.-H., Song J.-K., Song K.-I. (2013). Assessment of CO_2_ reduction of alkali-activated concrete. J. Clean. Prod..

[12] Turner L.K., Collins F.G. (2013). Carbon dioxide equivalent (CO_2_-e) emissions: A comparison between geopolymer and OPC cement concrete. Constr. Build. Mater..

[13] Dal Pozzo A., Carabba L., Bignozzi M.C., Tugnoli A. (2019). Life cycle assessment of a geopolymer mixture for fireproofing applications. Int. J. Life Cycle Assess..

[14] D’Alessandro A., Coffetti D., Crotti E., Coppola L., Meoni A., Ubertini F. (2020). Self-Sensing Properties of Green Alkali-Activated Binders with Carbon-Based Nanoinclusions. Sustainability.

[15] Cristelo N., Castro F., Miranda T., Abdollahnejad Z., Fernández-Jiménez A. (2021). Iron and Aluminium Production Wastes as Exclusive Components of Alkali Activated Binders—Towards a Sustainable Alternative. Sustainability.

[16] Onaizi A.M., Huseien G.F., Shukor Lim N.H.A., Tang W.C., Alhassan M., Samadi M. (2022). Effective Microorganisms and Glass Nanopowders from Waste Bottle Inclusion on Early Strength and Microstructure Properties of High-Volume Fly-Ash-Based Concrete. Biomimetics.

[17] McLellan B.C., Williams R.P., Lay J., van Riessen A., Corder G.D. (2019). Costs and carbon emissions for geopolymer pastes in comparison to ordinary portland cement. J. Clean. Prod..

[18] Ouellet-Plamondon C., Habert G. (2015). Life cycle assessment (LCA) of alkali-activated cements and concretes. Handbook of Alkali-Activated Cements, Mortars and Concretes.

[19] Robayo-Salazar R., Mejía-Arcila J., Mejía de Gutiérrez R., Martínez E. (2018). Life cycle assessment (LCA) of an alkali-activated binary concrete based on natural volcanic pozzolan: A comparative analysis to OPC concrete. Constr. Build. Mater..

[20] Salas D.A., Ramirez A.D., Ulloa N., Baykara H., Boero A.J. (2018). Life cycle assessment of geopolymer concrete. Constr. Build. Mater..

[21] Teh S.H., Wiedmann T., Castel A., de Burgh J. (2017). Hybrid life cycle assessment of greenhouse gas emissions from cement, concrete and geopolymer concrete in Australia. J. Clean. Prod..

[22] Metzger J.O., Eissen M. (2004). Concepts on the contribution of chemistry to a sustainable development. Renewable raw materials. Comptes Rendus Chim..

[23] Robayo-Salazar R.A., Valencia-Saavedra W., Mejía de Gutiérrez R. (2020). Construction and Demolition Waste (CDW) Recycling—As Both Binder and Aggregates—In Alkali-Activated Materials: A Novel Re-Use Concept. Sustainability.

[24] Bilek V., Sucharda O., Bujdos D. (2021). Frost Resistance of Alkali-Activated Concrete—An Important Pillar of Their Sustainability. Sustainability.

[25] Faridmehr I., Bedon C., Huseien G., Nikoo M., Baghban M. (2021). Assessment of Mechanical Properties and Structural Morphology of Alkali-Activated Mortars with Industrial Waste Materials. Sustainability.

[26] Maniya K., Bhatt M.G. (2010). A selection of material using a novel type decision-making method: Preference selection index method. Mater. Amp; Des..

[27] Fernández-Jiménez A., Palomo J.G., Puertas F. (1999). Alkali-activated slag mortars. Cem. Concr. Res..

[28] Lavanya G., Jegan J. (2015). Evaluation of relationship between split tensile strength and compressive strength for geopolymer concrete of varying grades and molarity. Int. J. Appl. Eng. Res.

[29] Choi Y., Yuan R.L. (2005). Experimental relationship between splitting tensile strength and compressive strength of GFRC and PFRC. Cem. Concr. Res..

[30] Davidovits J., Comrie D.C., Paterson J.H., Ritcey D.J. (1990). Geopolymeric concretes for environmental protection. ACI Concr. Inter..

[31] Bakharev T., Sanjayan J.G., Cheng Y.-B. (2003). Resistance of alkali-activated slag concrete to acid attack. Cem. Concr. Res..

[32] Pawlasova S., Skavara F. High-temperature properties of geopolymer materials. Proceedings of the Alkali Activated Materials-Research, Production and Utilization 3rd Conference.

[33] Krivenko P., Guziy S. Fire resistant alkaline portland cements. Proceedings of the Alkali Activated Materials Research, Production and Utilization 3rd Conference.

[34] Dolezal J., Skvara F., Svoboda P., Sulc R., Kopecky L., Pavlasova S., Myskova L., Lucuk M., Dvoracek K. Concrete based on fly ash geopolymers. Proceedings of the Alkali Activated Materials Research, Production and Utilization 3rd Conference.

[35] Fernando P.-T., João C.-G., Said J. (2010). Durability and Environmental Performance of Alkali-Activated Tungsten Mine Waste Mud Mortars. J. Mater. Civ. Eng..

[36] Bumanis G., Korjakins A., Bajare D. (2022). Environmental Benefit of Alternative Binders in Construction Industry: Life Cycle Assessment. Environments.

[37] Munir Q., Kärki T. (2021). Cost Analysis of Various Factors for Geopolymer 3D Printing of Construction Products in Factories and on Construction Sites. Recycling.

[38] Katarzyna B., Le C.H., Louda P., Michał S., Bakalova T., Tadeusz P., Prałat K. (2020). The Fabrication of Geopolymer Foam Composites Incorporating Coke Dust Waste. Processes.

[39] Łach M. (2021). Geopolymer Foams—Will They Ever Become a Viable Alternative to Popular Insulation Materials? A Critical Opinion. Materials.

[40] Alkhawaldeh A.A., Alhassan M.A., Elrefae A. A Case Study of Implementing Life Cycle Cost Analysis in Sustainability Assessment. International Arab Conference on Information Technology. Proceedings of the 2022 International Arab Conference on Information Technology (ACIT).

[41] PachecoTorgal F., Jalali S. (2011). Eco-Efficient Construction and Building Materials.

[42] Ploskas N., Papathanasiou J. (2018). Multiple Criteria Decision Aid.

